# Supplementary material for: Retusone A, a Guaiane-Type Sesquiterpene Dimer from Wikstroemia retusa and Its Inhibitory Effects on Histone Acetyltransferase HBO1 Expression
Source: Molecules. 2022 May 3;27(9):2909. doi: 10.3390/molecules27092909 (PMC9105026; doi:10.3390/molecules27092909)
Supplement: Supplementary file 1 [file molecules-27-02909-s001.zip › molecules-1681447-supplementary.pdf]

Retusone A, A Guaiane-Type Sesquiterpene Dimer from *Wikstroemia retusa* and Its Inhibitory Effects on Histone Acetyltransferase HBO1 Expression

Young Sook Yun [1,*], Tomomi Nakano [1], Haruhiko Fukaya [1], Yukio Hitotsuyanagi [1], Miho Nakamura [1], Megumi Umetsu [1], Nobuko Matsushita [1,†], Katsunori Miyake [1], Hiroyuki Fuchino [2], Nobuo Kawahara [2,‡], Fuki Moriya [1], Akihiro Ito [1], Yuji Takahashi [1] and Hideshi Inoue [1]

[1] School of Life Sciences, Tokyo University of Pharmacy and Life Sciences, 1432-1 Horinouchi, Tokyo 192-0392, Japan

[2] Research Center for Medicinal Plant Resources, National Institutes of Biomedical Innovation, Health and Nutrition, Tsukuba 305-0843, Japan

[*] Correspondence: yun@toyaku.ac.jp; Tel.: +81-42-676-5319

[†] Present Address: School of Life and Enviromental Science, Azabu University, 1-17-71 Fuchinobe, Chuo-ku, Sagamihara-shi 252-5201, Japan; matsushita@azabu-u.ac.jp

[‡] Present Address: The Kochi Prefectural Makino Botanical Garden, 4200-6, Godaisan, Kochi City 781-8125, Japan; kawahara@makino.or.jp

Table S1. Crystal diffraction of **1**--S2
Table S2. Energy analysis for 2*S*,7*R*,14*S*,7′*R*,14′*S*-**1**---------------------------------S3
Figure S1. Lowest energy conformer for 2*S*,7*R*,14*S*,7′*R*,14′*S*-**1**--------------------S3
Figure S2. UV spectrum of **1**---S4
Figure S3. IR spectrum of **1**--S4
Figure S4. ^{1}H-NMR spectrum (500 MHz, $CDCl_3$) of **1**-----------------------------S5
Figure S5. ^{13}C-NMR spectrum (125 MHz, $CDCl_3$) of **1**---------------------------S6
Figure S6. ^{1}H-^{1}H COSY spectrum of **1**--- S7
Figure S7. HMQC spectrum of **1**-- S7
Figure S8. HMBC spectrum of **1**---S8
Figure S9. MS chart of **1**-- S8
Figure S10. ^{1}H-NMR spectrum (500 MHz, $CDCl_3$) of **2**----------------------------S9
Figure S11. ^{13}C-NMR spectrum (125 MHz, $CDCl_3$) of **2**--------------------------S9
Figure S12. MS chart of **2**--S10

Table S1. Crystallographic data for **1**.

Formula	$C_{31}H_{38}O_4$
$D_{calc.}$/ g cm^{-3}	1.215
μ/mm^{-1}	0.079
Formula Weight	474.61
Colour	colourless
Shape	plate
Size/mm^3	0.53×0.14×0.09
T/K	90
Crystal System	monoclinic
Flack Parameter	-1.0(6)
Hooft Parameter	-0.9(6)
Space Group	
a/Å	22.460(6)
b/Å	5.0621(13)
c/Å	24.319(6)
α/°	90
β/°	110.160(3)
γ/°	90
V/Å^3	2595.5(11)
Z	4
Z'	1
Wavelength/Å	0.71073
Radiation type	MoK$_\alpha$
Θ_{min}/°	1.784
Θ_{max}/°	27.555
Measured Refl's.	36724
Indep't Refl's	5968
Refl's I≥2 σ(I)	4867
R_{int}	0.0777
Parameters	321
Restraints	1
Largest Peak	0.342
Deepest Hole	-0.236
GooF	1.036
wR_2 (all data)	0.1265
wR_2	0.1173
R_1 (all data)	0.0723
R_1	0.0530

Single colourless plate crystals of **1** slow crystallization from a methanol solution at room temperature. A suitable crystal with dimensions 0.53 × 0.14 × 0.09 mm^3 was selected and the crystal was mounted on a MicroMount using perfluoropolyether oil on a Bruker SMART APEX II ULTRA diffractometer. The crystal was kept at a steady T = 90 K during data collection. The structure was solved with the ShelXT 2014/5 solution program[1] using dual methods and by using Olex2 as the graphical interface. The model was refined with XL using full matrix least squares minimisation on $\boldsymbol{F^2}$.

Table S2. Calculated Relative Energies (kcal/mol) and Populations (%) of the Three Lowest Energy Conformers of **1**.

Conformer	ΔG	P(%)[b]
**a**	0.00 [a]	48.8
**b**	0.49	21.2
**c**	0.79	12.9
**d**	0.88	11.0
**e**	1.23	6.1

[a] Calculated relative energies to **a** with ΔG = −944642.89839 kcal/mol at the B3LYP/6–311+G (2df, 2p) level in MeOH.
[b] Boltzmann population at T = 298 K and 1 atm.

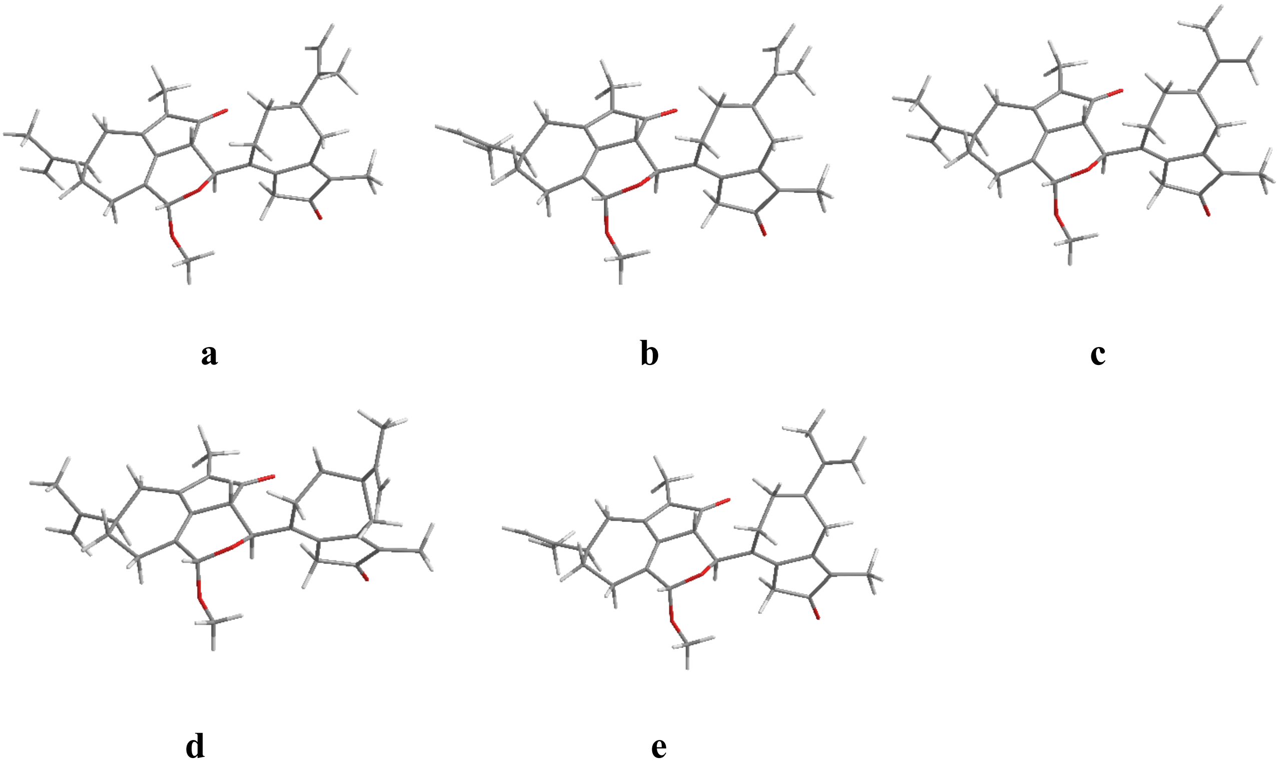

Figure S1. B3LYP/6–311+ G (2df,2p) optimized lowest energy conformers for **1**.

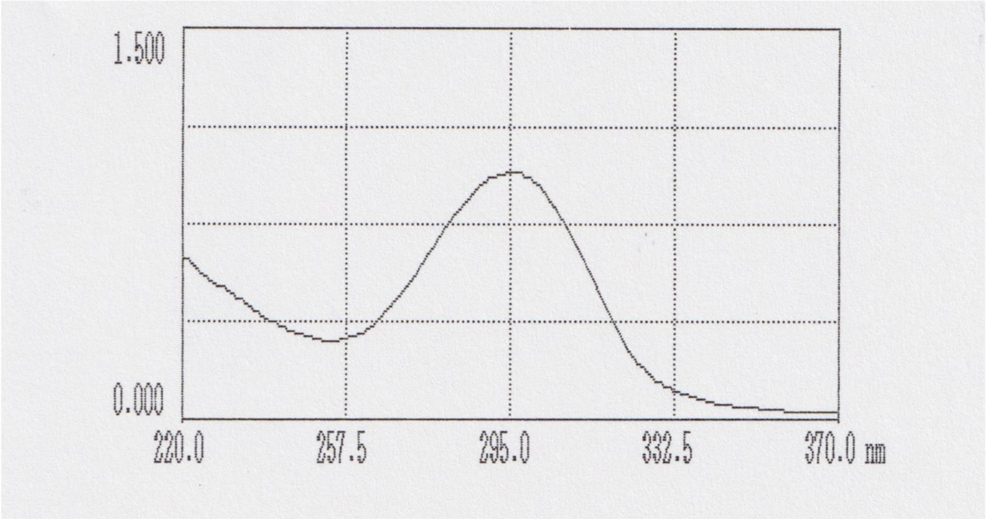

Figure S2. UV spectrum of **1**.

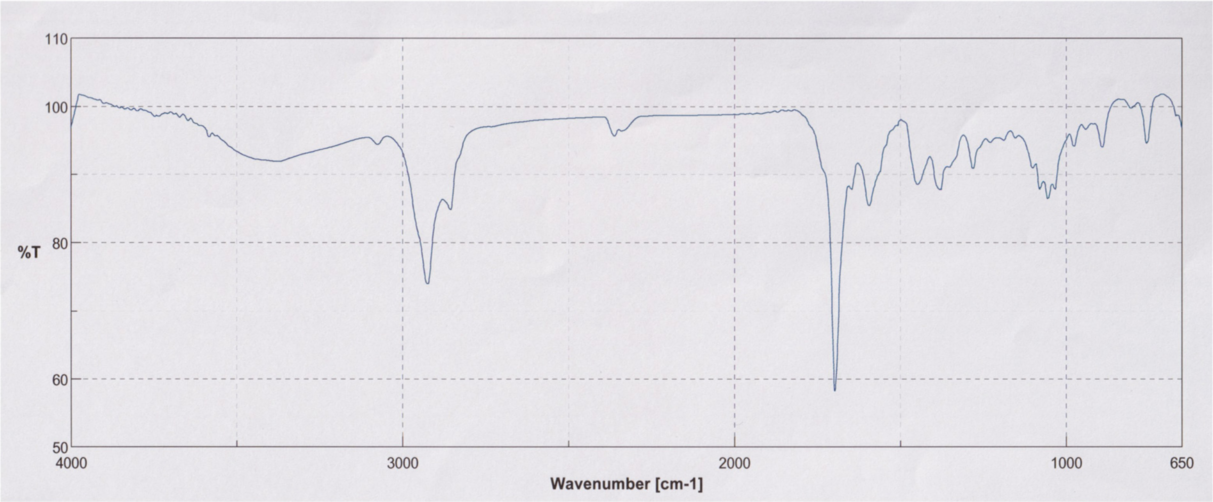

Figure S3. IR spectrum of **1**.

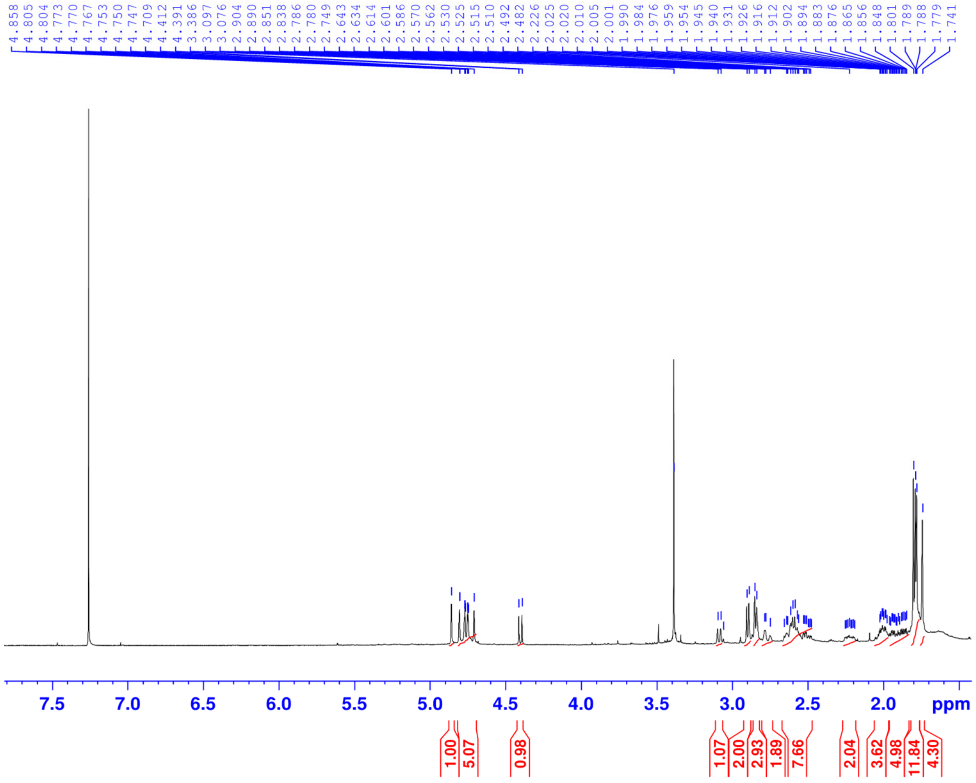

Current Data Parameters
NAME Nov12-2020-Bunshi
EXPNO 20
PROCNO 1

F2 - Acquisition Parameters
Date_ 20201112
Time 17.15
INSTRUM spect
PROBHD 5 mm PABBO BB/
PULPROG zg30
TD 65536
SOLVENT CDCl3
NS 8
DS 4
SWH 10000.000 Hz
FIDRES 0.152588 Hz
AQ 3.2767999 sec
RG 190.86
DW 50.000 usec
DE 6.50 usec
TE 300.0 K
D1 1.00000000 sec
TD0 1

======== CHANNEL f1 ========
SFO1 500.2330891 MHz
NUC1 1H
P1 11.00 usec
PLW1 27.00000000 W

F2 - Processing parameters
SI 65536
SF 500.2300126 MHz
WDW EM
SSB 0
LB 0.30 Hz
GB 0
PC 1.00

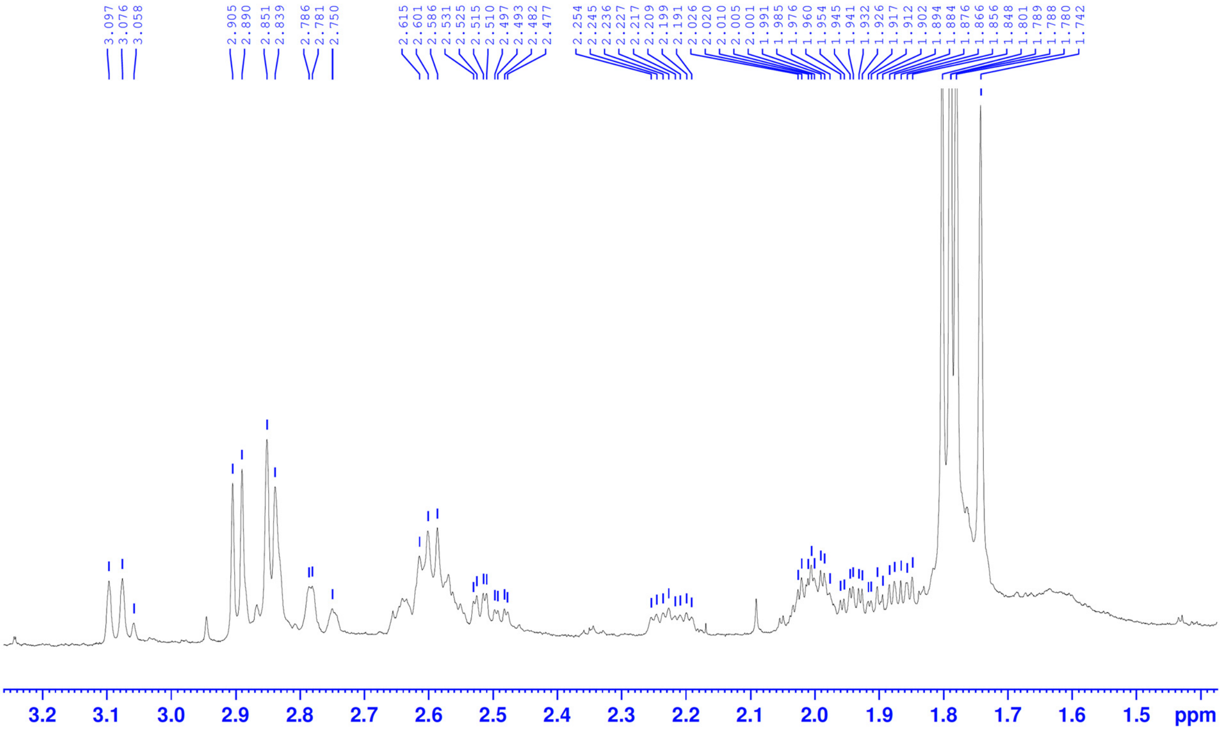

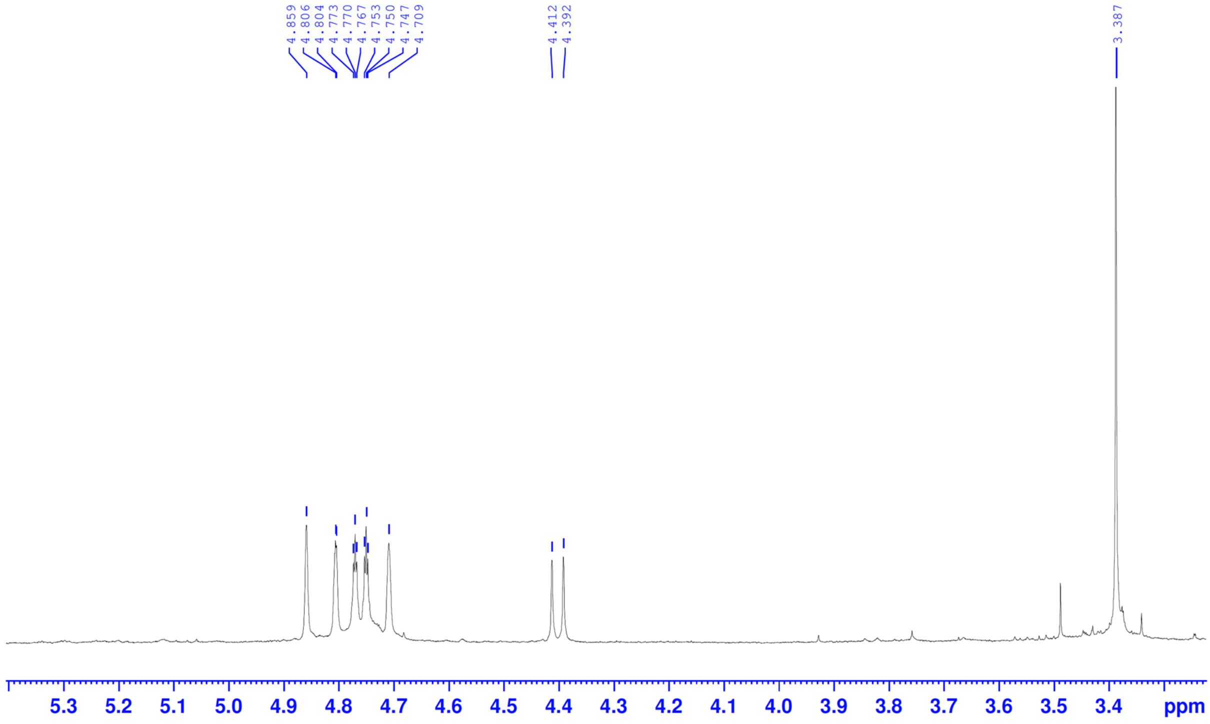

Figure S4. ^{1}H NMR spectrum (500 MHz, $CDCl_3$) of **1**.

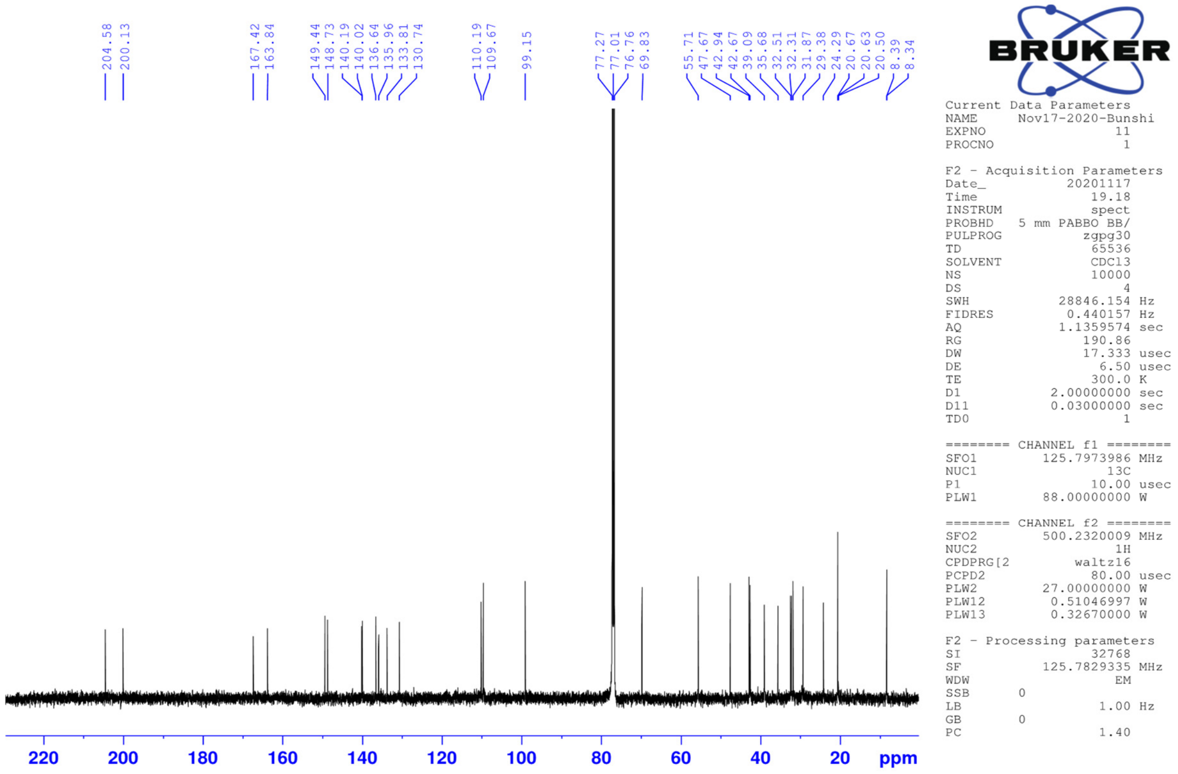

Figure S5. ^{13}C NMR spectrum (125 MHz, $CDCl_3$) of **1**.

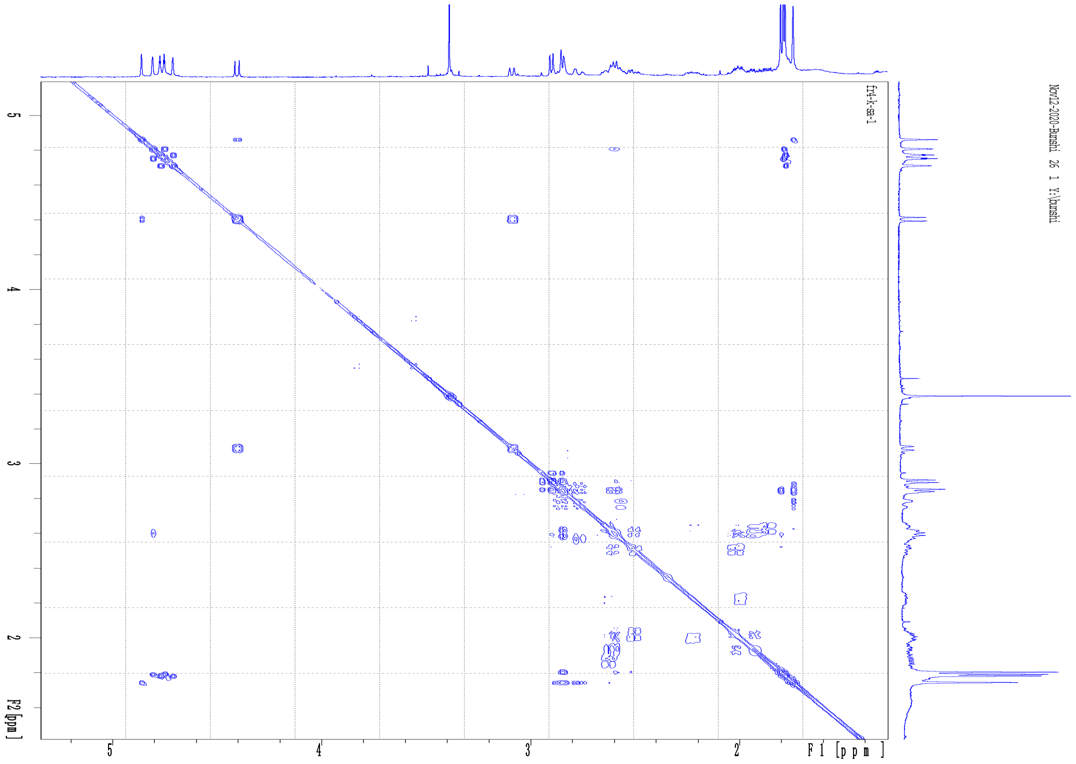

Figure S6. ^{1}H-^{1}H COSY spectrum of **1**.

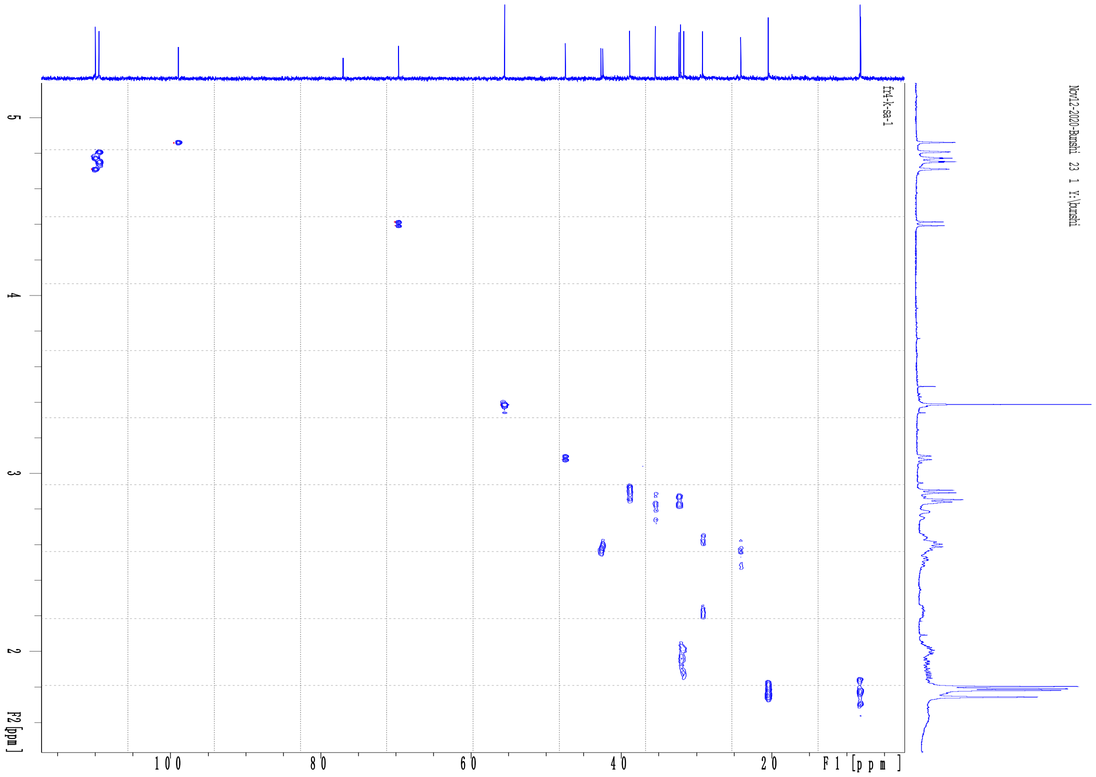

Figure S7. HMQC spectrum of **1**.

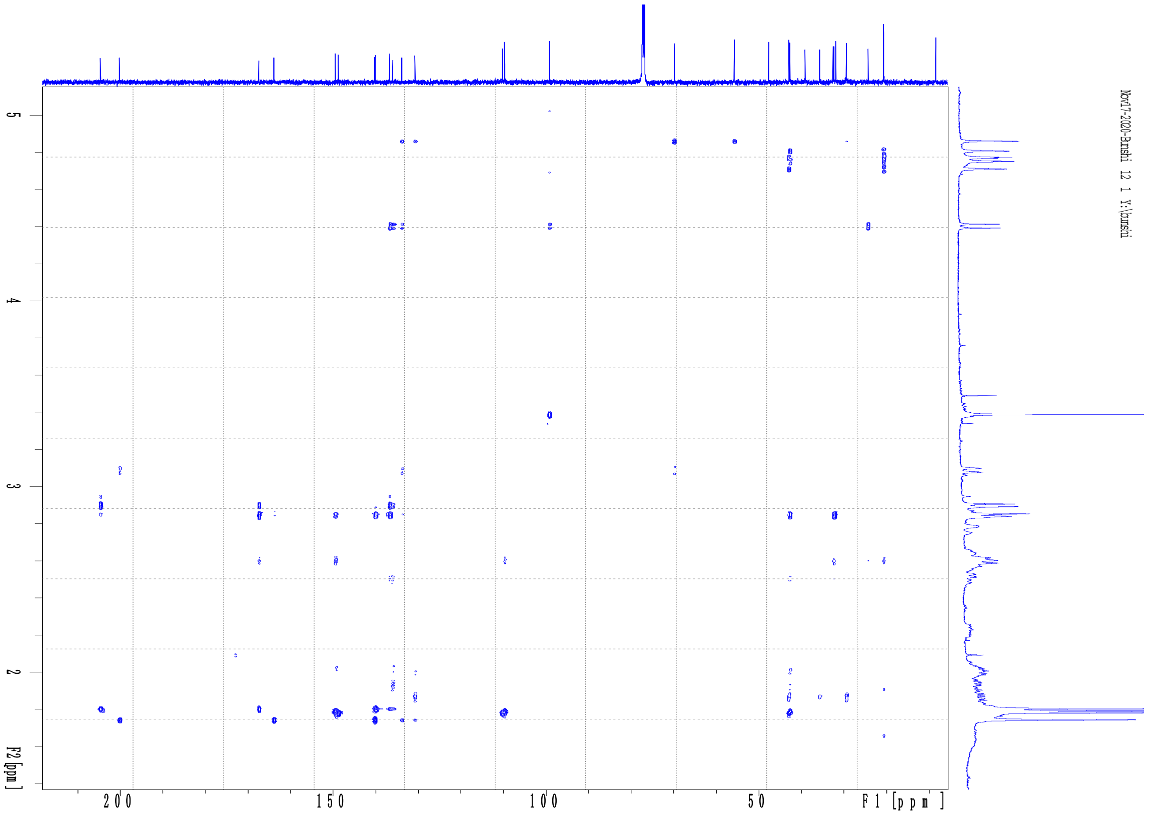

Figure S8. HMBC spectrum of **1**.

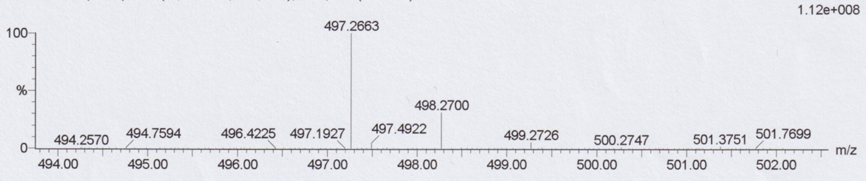

Elemental Composition Report **Page 1**

Single Mass Analysis
Tolerance = 10.0 mDa / DBE: min = -1.5, max = 300.0
Element prediction: Off
Number of isotope peaks used for i-FIT = 3

Monoisotopic Mass, Even Electron Ions
6 formula(e) evaluated with 1 results within limits (up to 50 closest results for each mass)
Elements Used:
C: 0-300 H: 0-1000 O: 4-4 Na: 1-1
kessyou
M-17424 138 (1.119) AM2 (Ar,22000.0,0.00,0.00); ABS; Cm (137:175)

	Mass	Calc. Mass	mDa	PPM	DBE	i-FIT	Norm	Conf(%)	Formula
Minimum:					-1.5				
Maximum:			10.0	50.0	300.0				
	497.2663	497.2668	-0.5	-1.0	12.5	680.4	n/a	n/a	C31 H38 O4 Na

Figure S9. HR-ESI-MS chart of **1**.

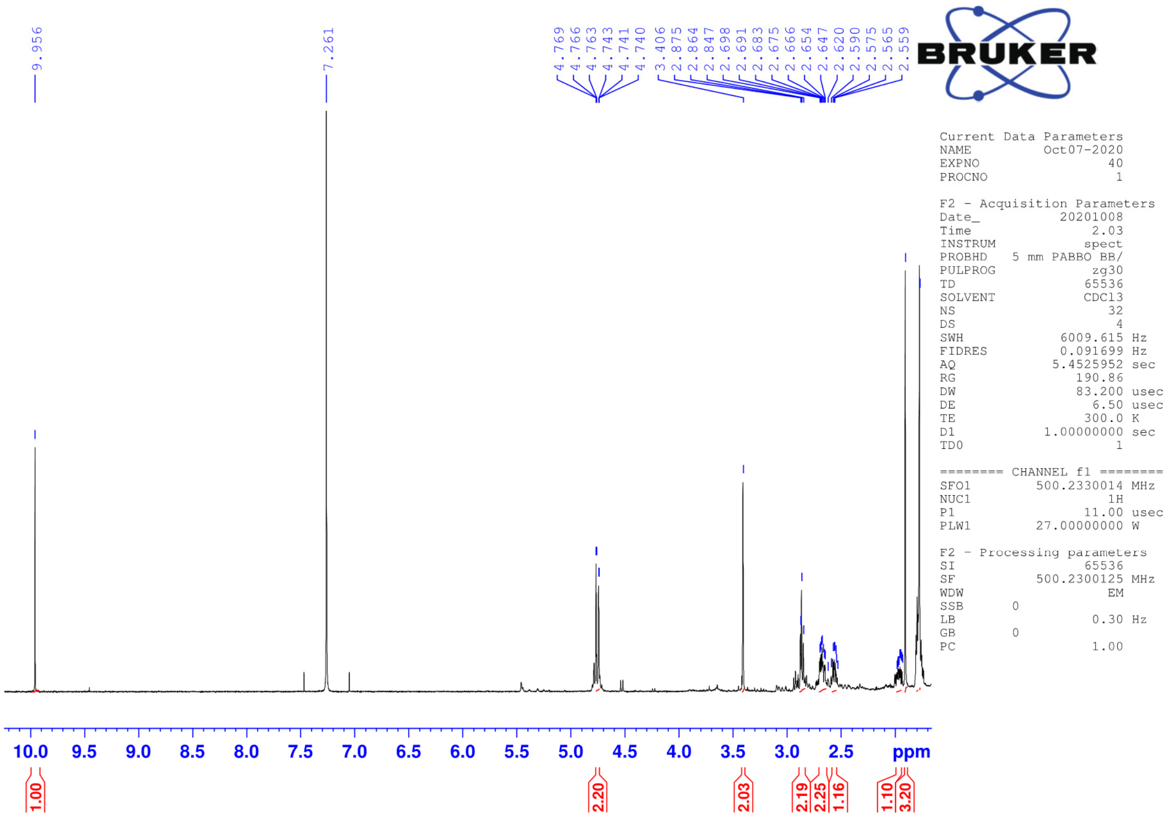

Figure S10. ^{1}H NMR spectrum (500 MHz, $CDCl_3$) of **2**.

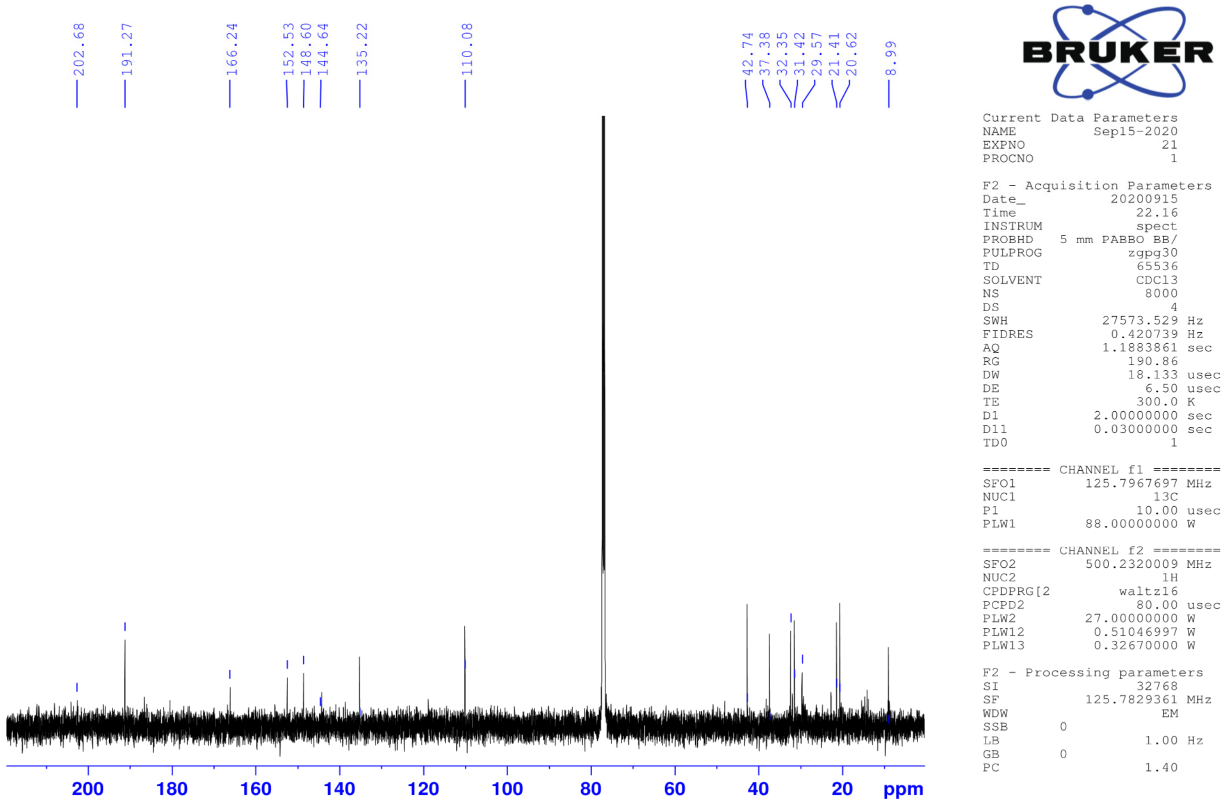

Figure S11. ^{13}C NMR spectrum (125 MHz, $CDCl_3$) of **2**.

Elemental Composition Report

Page 1

Single Mass Analysis
Tolerance = 10.0 mDa / DBE: min = -1.5, max = 300.0
Element prediction: Off
Number of isotope peaks used for i-FIT = 3

Monoisotopic Mass, Even Electron Ions
3 formula(e) evaluated with 1 results within limits (up to 50 closest results for each mass)
Elements Used:
C: 1-300 H: 1-1000 O: 2-2 Na: 1-1
fr3-3-2-1
M-17259 265 (2.133) AM2 (Ar,22000.0,0.00,0.00); ABS; Cm (230:267)

1: TOF MS ES+
5.63e+007

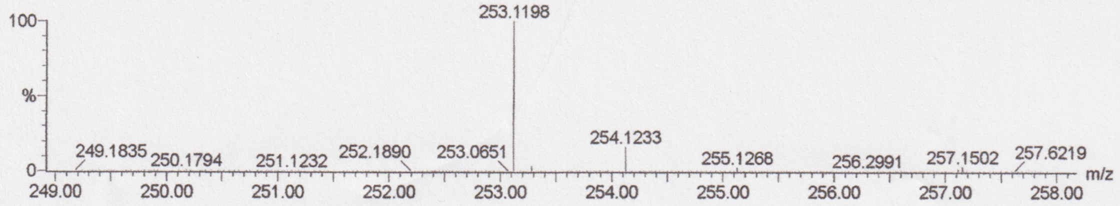

Minimum:				-1.5				
Maximum:		10.0	50.0	300.0				
Mass	Calc. Mass	mDa	PPM	DBE	i-FIT	Norm	Conf(%)	Formula
253.1198	253.1204	-0.6	-2.4	6.5	1001.6	n/a	n/a	C15 H18 O2 Na

Figure S12. HR-ESI-MS chart of **2**.
